# The New Ohio Dental Law, (House Bill No. 151)

**Published:** 1902-07-15

**Authors:** W. S. McKinnon, Carl L. Nippert

**Affiliations:** Speaker of the House of Representatives; President of the Senate


### THE NEW OHIO DENTAL LAW.

[House Bill No. 151.]

AN ACT

To amend sections 4404 and 6991 of the Revised Statutes of Ohio.

Be it enacted by the General Assembly of the State of Ohio:

Section 1. That sections 4404 and 6991 of the Revised Statutes of Ohio be so amended as to read as follows:

Sec. 4404. From and after June 1, 1902, it shall be unlawful for any person to practice dentistry in this State, unless such person shall have first obtained a certificate of qualification issued by the State board of dental examiners of this State, as hereinafter provided.

1. A board of dental examiners, to consist of five practicing dentists, not members of dental college faculties, resident in this State, is hereby created, whose duty it shall be to carry out the purposes and to enforce the provisions of this act. The members of the first board of dental examiners under the provisions of this act shall be appointed by the governor of the State on or before the first day in June, 1902. The term for which members of said board shall be appointed shall be three years, and until their successors shall be duly appointed and qualified, and no person shall be appointed for or serve to exceed two terms in succession. All vacancies in said board caused by expiration of term, or otherwise, shall be filled by appointment by the governor of the State.

2. Said board shall have power to make reasonable rules and regulations for the purpose of carrying out and enforcing the provisions of this act. It shall choose one of its members president, one secretary and one treasurer; and shall hold two regular meetings in the city of Columbus, on the last Tuesday in June and November, in each year, and at such other times as may be deemed necessary by said board. A majority of said board shall at all times constitute a quorum thereof for the transaction of business, but a less number may adjourn from time to time. The board shall keep full minutes of all of its proceedings, and a full register of all persons licensed and certified as dentists by said board, which shall be public records, and at all reasonable times open to inspection as such. The board shall render an annual itemized report of all moneys received and disbursed, also a summary of all official acts to the governor of the State, upon the first day of January each and every year. A transcript of any of the entries in such minutes and register, certified by the secretary, under the seal of said board, shall at all times and places be competent evidence of the facts therein stated. The members of the board shall have power to administer oaths, and the board shall have power to hear testimony in all matters relating to the duties imposed upon it by law.

3. Any and all persons who shall desire to practice dentistry in this State after June 1, 1902, except such persons as are legally practicing dentistry, as defined in this act in this State at the time of the passage of this act, shall file application in writing with the secretary of said board of dental examiners for examination or license or both, and at the time of making such application shall pay to the secretary of said board a fee of twenty dollars; except such person or persons who are not required to take the State board examination, shall pay a license fee of ten dollars; and each applicant shall present himself before said board at its first meeting after filing his application for examination or license by said board. The applicant for examination shall present a diploma from some legally chartered dental college and pass a satisfactory examination as hereinafter described. Upon a unanimous vote of the board, said board may excuse from examination an applicant holding a license to practice in some State requiring a diploma and examination, upon the payment of the examination fee. The board shall excuse from examination all graduates of dental colleges of this State up to and including the June, 1905, session of the board; also any person or all persons who has or have been the proprietor or proprietors of a dental office or a place of performing dental work in this State, continuously since January 1, 1893. Such person or persons shall be licensed and registered upon application and paying such license fee as is herein provided. The board shall admit to examination, during the years 1902 and 1903, any person who is a legal resident of this State, and has been a student of dentistry, under a preceptor, for twelve months prior to the passage of this act. The examination shall consist of practical demonstrations and written or oral tests, or both, at the option of the board, and shall include the following subjects, to-wit: anatomy, physiology, chemistry, materia medica, therapeutics, metallurgy, histology, pathology, bacteriology, prosthetic, operative and surgical dentistry. All persons successfully passing such examinations, or as above provided, and of good moral character, shall be registered and licensed by the said board of dental examiners, and shall receive a certificate of such registration and license duly authenticated by the seal and signature of the president and secretary of said board. In no case shall the examination fee be refunded. But said applicant may be re-examined within twelve months without any additional fee.

4. Every person receiving such a certificate of registration and license as dentist shall, before engaging in the practice of dentistry in this State, place and retain in place while engaged in the practice of dentistry in this State, such certificate of registration and license in a conspicuous position at his place of business, in such manner as to be easily seen and read. The certificate of the secretary of said board of dental examiners, under the seal of said board, stating that any person is a registered and licensed dentist, shall be prima facie evidence that such person is entitled to practice dentistry in this State.

Sec. 6991. All persons shall be said to be practicing dentistry within the meaning of this act who shall be known as a manager, proprietor, operator or conductor of a dental office or a place of performing dental work, or who shall for a fee, salary or other reward paid, or to be paid, either to himself or to another person, perform dental operations of any kind, treat diseases or lesions of human teeth or jaws, or attempt to correct mal-positions thereof. But nothing contained in this act shall be taken to apply to acts of bona fide students of dentistry, done in the pursuit of clinical knowledge in the clinic rooms of a regularly organized dental college which is approved by the board of dental examiners of this State, or under the direct supervision of a preceptor who is a licensed dentist in this State, during the regular vacation intervals of a college course, said student being matriculated in, and pursuing consecutive courses of study in a dental college recognized by the board of dental examiners of the State; or the acts of legally qualified physicians and surgeons, unless practicing dentistry as a specialty.

1. Out of the funds coming into the possession of the board as above specified, the members of said board may each receive a compensation in the sum of ten dollars for each day actually engaged in the duties of their office as such examiners, and a mileage of three cents per mile for all distance necessarily traveled in going to and coming from the meetings of the board. Said board may, with the consent of the prosecuting attorney of any county, employ and compensate out of said fund special counsel to assist in the prosecution in the courts of said county and the supreme court any offense alleged to have been committed under the provisions of this act in such county. Said expenses shall be paid from the fees and assessments received by the board under the provisions of this act, and no part of the salary and other expenses of the board shall ever be paid out of the State treasury. All moneys received in excess of the said per diem allowance and mileage, as above provided for, shall be held by the treasurer of said board as a special fund for other expenses of said board and carrying out provisions of this act, and said treasurer shall give such bond as the board shall from time to time direct.

2. Any person who shall violate any of the provisions of this act shall be guilty of a misdemeanor, and upon conviction thereof may be fined not less than fifty dollars nor more than one hundred dollars, or be confined not less than ten days nor more than one month in the county jail, or both. A second conviction shall be punished by the maximum penalties presented in this act. All fines thus received shall be paid into the common school fund of the county in which such conviction takes place. It is hereby made the duty of the prosecuting attorney of each county in the State to prosecute every case to final judgment whenever his attention shall be called to a violation of the provisions of this act.

3. Any person who shall knowingly or falsely claim or pretend to have or hold a certificate of registration, or who shall falsely and with intent to deceive the public, claim or pretend to be a registered or licensed dentist, not being such a registered or licensed dentist, shall be guilty of a misdemeanor, and shall be fined the maximum penalties provided in this act.

4. The board of examiners created by this amended act may sue or be sued, and in all actions brought by or against it, it shall be made a party under the name of the board of dental examiners of the State of Ohio, and no suit shall abate by reason of any change in the membership of said board.

Section 2. Said original sections 4404, 4404*a* and 6991 of the Revised Statutes of Ohio are hereby repealed.

Section 3. This act shall take effect and be in force from and after its passage.

W. S. McKinnon,
*Speaker of the House of Representatives.*
Carl L. Nippert,
*President of the Senate.*

Passed April 29, 1902. 204G

